# Laboratory In-Situ Production of Autochthonous and Allochthonous Fluorescent Organic Matter by Freshwater Bacteria

**DOI:** 10.3390/microorganisms9081623

**Published:** 2021-07-29

**Authors:** Bethany G. Fox, Robin M. S. Thorn, Darren M. Reynolds

**Affiliations:** Centre for Research in Biosciences, University of the West of England, Bristol BS16 1QY, UK; Bethany.Fox@uwe.ac.uk (B.G.F.); Robin2.Thorn@uwe.ac.uk (R.M.S.T.)

**Keywords:** fluorescent organic matter, autochthonous, allochthonous, excitation emission matrix fluorescence spectroscopy, environmental microbiology

## Abstract

This work investigates the origin and range of fluorescent organic matter (FOM) produced in-situ by environmentally sourced freshwater bacteria. Aquatic FOM is an essential component in global carbon cycling and is generally classified as either autochthonous, produced in-situ via microbial processes, or allochthonous, transported into aquatic systems from external sources. We have demonstrated that, within laboratory model systems, environmentally sourced mixed microbial communities and bacterial isolates can produce and/or export FOM associated with both autochthonous and allochthonous material. This study focuses on fluorescence peak B, T, M, C and C+, exploring (1) the cellular nature of FOM produced, (2) FOM exported as extracellular material into the water column and (3) the impact of physical cell lysis on FOM signature. For the laboratory model systems studied, Peak T fluorescence is retained within bacterial cells (>68%), while Peak C fluorescence is mainly observed as extracellular material (>80%). Peak M is identified as both cellular and extracellular FOM, produced by all isolated freshwater microorganisms investigated. The origin of Peak C+ is postulated to originate from functional metabolites associated with specific microorganisms, seen specifically within the *Pseudomonas* sp. monoculture here. This work challenges the binary classification of FOM as either allochthonous or autochthonous, suggesting that FOM processing and production occurs along a dynamic continuum. Within this study, fluorescence intensity data for the environmental bacteria isolate monocultures are presented as enumeration corrected data, for the first time providing quantitative fluorescence data per bacterial colony forming unit (cfu). From this, we are able to assess the relative contribution of different bacteria to the autochthonous FOM pool and if this material is cellular or extracellular.

## 1. Introduction

Dissolved organic matter (DOM) is an essential component of global biogeochemical cycles. DOM is a highly complex chemical composite [1], and quantitative chemical analyses of DOM composition requires the use of high resolution quantitative techniques, such as Fourier-Transform Ion-Cyclotron-Resonance Mass Spectrometry (FTICR-MS) coupled with High-Performance Liquid Chromatography (HPLC) [2,3,4,5]. Optical techniques for the interrogation of DOM characteristics and dynamics are increasingly used by researchers due to the ease of data collection through time and space [1,6]. Although optical techniques, such as specific UV absorbance (SUVA_254_) and fluorescence spectroscopy, are not appropriate for all DOM components, they lend themselves to in-field use and high frequency monitoring, enabling in-situ real-time data with high temporal and spatial resolution [1,4]. This has led to extensive use of optical data for the investigation and monitoring of DOM in a variety of aquatic systems [3,6,7,8].

Fluorescent organic matter (FOM), the naturally fluorescent fraction of DOM, has been characterised across a range of aquatic systems [6,7,8,9,10,11]. This has led to the classification of FOM in aquatic environments as either autochthonous, produced in-situ via microbial processes, or allochthonous, transported into the system from external sources [6,12,13,14]. Within this binary classification, the ‘microbially-derived’ autochthonous material contributes to fluorescence peaks B, T and M, whereas the ‘terrestrially-sourced’ allochthonous material, considered to be complex high molecular weight FOM [15,16,17], contributes to fluorescence peaks C, C+ and A [4,7,18,19]. Although the classification of high molecular weight material varies greatly throughout DOM literature, low molecular weight material is consistently identified as being <1 kDa [15,20,21,22,23,24,25,26]. In line with the literature and the recent finding that the majority (75%) of aquatic fluorophores are <1.8 kDa [20], this paper refers to molecules > 1 kDa as high molecular weight and those <1 kDa as low molecular weight.

Classifying FOM in this way has enabled the study of the variable temporal and spatial detection of these fluorescent signatures within aquatic systems, as indicators of water quality [6]. Freshwater research has mainly focussed on the use of Peak T fluorescence as an in-situ indicator of the presence of microorganisms [27,28], and more recently how this can be used to infer microbial activity within freshwater systems [29,30,31]. Microbial activity has long been studied within marine sciences due to the importance of the microbial-carbon pump and long-term deep ocean carbon storage [11,32,33,34]. FOM research in this area has explored the ability of marine microorganisms to produce and process a range of FOM in simulated laboratory models, but has focussed on recalcitrant carbon (e.g., peaks C and M) rather than the labile fraction (e.g., peaks T and B) [5,35,36,37].

Although there has been some exploration of the autochthonous origin of microbial FOM outside of marine research, this has been limited to the exploration of single bacterial species [38] or has used laboratory based-models that employ reference strain microbial inoculums [39,40]. This has demonstrated the ability of microbes to produce a wide range of FOM in-situ, providing an insight into the potential use of Peak T fluorescence as a proxy of microbial activity or the presence of bacteria. However, the production and release of FOM by bacteria present in freshwater systems and its utilisation as a carbon source has not been fully explored. This knowledge gap is vital for understanding the extent that freshwater systems and their microbial communities’ impact global carbon cycling [41,42]. While knowledge of the marine microbial-carbon pump has increased over the past two decades, the role that freshwater systems play in global carbon cycling is key for improved climatic modelling and predictions [41,43,44].

The aim of this study was to investigate the in-situ production of FOM by isolated freshwater microorganisms. Furthermore, this work challenges current assumptions that freshwater FOM is perceived to be either autochthonous or allochthonous in origin. An improved understanding of the role of environmental microorganisms in the production of FOM in freshwaters will help address current knowledge gaps regarding the importance of surface freshwater systems within global carbon cycling.

An environmental freshwater sample was enriched and grown to assess the role of mixed microbial communities in the production of FOM. Samples were analysed using fluorescent spectroscopy to produce Excitation Emission Matrices (EEM) spectra, which enabled investigation of the resultant FOM. The use of the microbial community within a model system provides a snapshot of the potential community and FOM production from this system. Bacteria from this environmental freshwater sample were also isolated, cultured and subjected to the same analysis as the mixed freshwater microbial community. The environmentally derived bacterial monocultures allowed for a more in-depth exploration of the FOM production at an individual environmental isolate level. All culture samples were fractioned prior to analysis to elucidate the microbial origin of FOM production at a cellular level in freshwater systems, i.e., whether FOM is associated with cellular material or is produced and then exported by microorganisms into the water system.

## 2. Materials and Methods

### 2.1. Environmental Freshwater Sampling Location and Collection

A water sample was collected from an environmental freshwater body located at the University of the West of England, Bristol (UWE), UK (51°29′56″ N, 2°32′39″ W). Water was collected at a depth of ~30 cm from the surface, and ~3 m from the edge of the waterbody. Samples were collected using a five litre HDPE container, cleaned with a 1% Virkon ™ (Antec International Ltd., Sudbury, UK) solution, and then rinsed thoroughly with deionised water to prevent chemical or biological contamination. The container was rinsed three times with sample water immediately prior to collection. Details of the physicochemical data for the freshwater body on the day of collection can be found in the Supplementary Materials (Table S1).

### 2.2. Environmental Freshwater Microbial Cultures

#### 2.2.1. Environmental Microbial Community Inoculum

The environmental freshwater sample (Section 2.1) was filtered at 11 µm (Millipore 11.0 µm Net Nylon filter, MilliporeSigma, Burlington, MA, USA) to remove large particulate matter, i.e., sediment, whilst retaining the microbial community. Aliquots of the filtered water sample (1 mL) were added to 9 mL of a minimal media, detailed in Section 2.3, giving a final sample volume of 10 mL. These samples were incubated overnight (24 h) at 30 °C. The resulting overnight cultures were then centrifuged for 10 min at 5000× *g* and washed three times in osmotically stable ¼ strength Ringer’s solution (Oxoid Ltd., Basingstoke, UK), to remove media/supernatant and organic matter. The cell pellets were then resuspended in 10 mL of the minimal media and used as the microbial inoculum for FOM production experiments.

#### 2.2.2. Isolation of Environmental Bacterial Strains

Aliquots (100 mL) of the environmental freshwater sample were vacuum filtered through sterile 0.2 µm filters (Whatman^®^ 0.2 µm nitrocellulose membrane filters, GE Healthcare, Chalfont St Giles, UK). These filters were placed on selective and differential agar plates and incubated overnight (24 h) at 30 °C to culture a range of environmental bacteria. Twelve single colonies were identified from the plates, and sub-cultured on nutrient agar plates for 24 h at 30 °C: five colonies were taken from the R2A agar (Oxoid Ltd., Basingstoke, UK) plates; four colonies taken from the Brilliance ™ *E. coli*/Coliform (Oxoid Ltd., Basingstoke, UK) plates, which were presumptive *E. coli* and presumptive coliforms; and three colonies selected from the Difco ™ *Pseudomonas* Isolation Agar (Thermo Fisher Scientific Inc., Kent, DE, USA) plates, two of which were presumptive *Pseudomonas sp*.

The 12 unknown environmental bacterial isolates were subsequently subjected to an identification process using a BiOLOG MicroStation ™ (BioTek Instruments, Winooski, VT, USA) [45]. Prior to the BiOLOG ™ inoculum preparation, a Gram stain [46] was conducted to ensure the correct inoculating fluid and well-plates were used for identification. A catalase test [47] was also undertaken for the 12 species to narrow the species library search further. Five of the environmental bacterial isolates were identified with probability >90% to at least the Genus level. These identifications were verified by analytical profile index (API) tests (bioMérieux SA, Marcy-l’Étoile, France), biochemical tests for rapid identification of bacteria.

The five environmental bacterial isolates that were successfully identified were: *Bacillus* sp. (Gram positive, catalase positive); *Enterobacter* sp. (Gram negative, catalase positive); *Escherichia coli* (Gram negative, catalase positive); *Pseudomonas* sp. (Gram negative, catalase positive); and *Staphylococcus* sp. (Gram positive, catalase positive).

### 2.3. Minimal Media Composition

A non-fluorescent minimal medium, containing no proteinaceous material, was used to promote growth of the cultures within our laboratory model system. The basal medium contained a 0.2% *v*/*v* glucose solution, with sources of phosphate, nitrogen, sodium and magnesium [48], CaCl_2_ (final concentration 0.035% *v*/*v*) and trace elements (concentration 0.1% *v*/*v*) [49]. This was prepared as described by Fox et al. (2017).

### 2.4. Bacterial Culture Analysis

Overnight 20 mL cultures (24 h), for both the environmental microbial community inoculum and the environmental bacterial isolate monocultures, were incubated at 30 °C with shaking (150 rpm). The overnight cultures were then fractionated to provide information about cellular and extracellular FOM and to investigate the contribution of cell lysis to the FOM signal [38]. The overnight cultures were fractionated into three sub-sample types; supernatant, resuspended cells and lysed cells. The methods for the sample fractionation are detailed in our previous work [39]. No chemicals were used in the fractionation process to ensure the integrity of the fluorescence properties of the sample. This entire process was repeated three times, with each biological replicate also being run in triplicate. Sterile media (without inoculation) were used as negative controls for all experiments to ensure fluorescent signatures were not derived from the media, sample storage or sample processing.

The overnight monocultures for the five environmental bacterial isolates, detailed in Section 2.4, were enumerated. The bacterial suspensions were serially diluted in ¼ strength Ringer’s solution prior to being plated onto nutrient agar (Oxoid Ltd., Basingstoke UK) using a Spiral Plater (Whitley Automated Spiral Plater, Don Whiteley Scientific, Bingley, UK). Plates were incubated at 30 °C for 24 h, and colonies counted to provide numbers of viable colony forming units (cfu mL^−1^). Enumeration for each bacterial isolate was repeated three times, with each biological replicate also being enumerated in triplicate.

### 2.5. Fluorescence Measurements

Fluorescence excitation emission matrices (EEMs) of the overnight culture subsamples were collected using an Aqualog^®^ (Horiba Ltd., Kyoto, Japan). Bacterial supernatant samples were filtered using sterile Minisart^®^ 0.2 μm cellulose syringe filters (Sartorius Stedim Biotech, Goettingen, Germany) to guarantee that all cells were removed before being analysed. Cellular samples (resuspended and lysed cells) were not filtered prior to spectroscopic analysis to ensure sample integrity [39,50]. The EEM scan parameters were: λ_ex_ 240–500 nm at 1 nm steps, λ_em_ 247.88–829.85 nm in 1.16 nm steps, with an integration time of 500 ms. A micro quartz cuvette (1400 μL) with a 10 mm path-length was used throughout.

All fluorescence spectra were blank subtracted using sterile media, corrected for inner filter effects (for both excitation and emission wavelengths) and first and second order Rayleigh Scattering masked (±10 nm at λ_ex_ = λ_em_ and 2λ_ex_ = λ_em_) [8,39,40,51] within the Aqualog^®^ software (Horiba Ltd., Kyoto, Japan). All fluorescence intensity data were converted and reported in quinine sulphate units (QSU) (1 QSU = 1 µg^−1^ quinine sulphate) [19,39,40,52,53] via a custom script, written in Python^TM^ (Python Software Foundation, Wilmington, DE, USA).

#### 2.5.1. Fluorescence Data Analysis

A custom script, written in Python^TM^ (Python Software Foundation, Wilmington, DE, USA), was used to create the EEM maps and to undertake the peak picking for specific fluorescence regions of interest [39,40]. The data window of the EEMs was λ_ex_ 240–490 nm, λ_em_ 250–550 nm. EEM data were also investigated by employing parallel factor (PARAFAC) analysis [54] in Solo (Eigenvector Research Inc., Wenatchee, WA, USA) software, in conjunction with the MATLAB^®^ PLS-Toolbox (Mathworks, Natick, MA, USA).

Fluorescence intensity data for all sample fractions of the environmental bacterial isolate monocultures have been cell density normalised, obtained from viable counts (Section 2.4), whereby QSU is expressed as fluorescence intensity per 10^10^ cfu mL^−1^. To simplify the data and enhance visual interpretation, the data have been converted to log numbers as is standard practice for microbial counts. Consequently, monoculture fluorescence data are expressed as “Log Normalised QSU”. For the environmental microbial community culture, the fluorescence intensity data for all sample fractions are not normalised for cell density, due to the limitations of representatively culturing complete environmental bacterial communities.

The statistical significance of variation between fluorescence peak intensity data, obtained via peak picking, for the different sample fractions was undertaken using a two-way ANOVA. A paired t-test was conducted to determine if there was a significant difference between the extracellular (supernatant fraction) and the cellular (both resuspended cell and lysed cell fractions) locations of the fluorescence peak intensity data. A *p* value of <0.05 was regarded as significant. All statistics were performed in Prism 9 (GraphPad, San Diego, CA, USA).

## 3. Results

This work investigated the in-situ production of FOM by mixed environmental microbial communities and environmental bacterial isolates using a laboratory model system. The environmental microbial community culture and environmental bacterial isolates used here were obtained from the same water sample. The use of the microbial community within this model system provides a “snapshot” of the FOM production within this system, whilst the bacterial isolates allow for a more in-depth exploration of the FOM production at an individual environmental isolate level within the laboratory model system.

PARAFAC analysis was performed on the EEM datasets obtained from the freshwater model system. This analysis was unable to provide a robust model, CORCONDIA > 90% [55], for the microbial community cultures data (*n* = 234) nor the bacterial isolates data (*n* = 268). This is likely to be due to the dominance and ubiquitous nature of certain fluorescence peaks, namely peaks T and C. Subsequently, all EEM data obtained from the overnight cultures were subjected to peak picking, an established method for spectral analysis [56]. The fluorescence peaks interrogated are detailed in Table 1.

All peaks detailed in Table 1 were identified within the microbial community culture. However, the intensity of Peak B within the monocultures was seen to either decline during the 24 h culture period, or likely to increase due to the interference of the Peak T region. The ubiquitous presence and increase in intensity of the commonly identified fluorescence peaks T, C, C+ and M will be the focus of the work here.

### 3.1. Environmental Microbial Community Cultures

**Figure 1:**
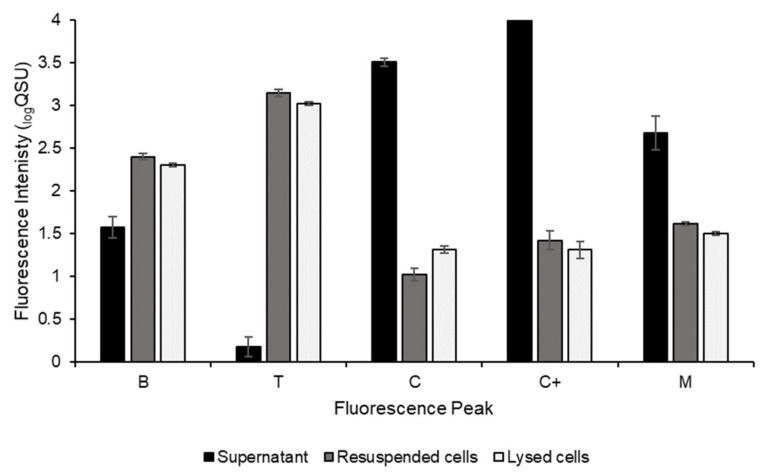
Apportionment of observed fluorescent organic matter produced by an environmental microbial community, derived from an environmental freshwater sample, cultured for 24 h at 30 °C. Fluorescence intensity (QSU, 1 QSU = 1 µg L^−1^ quinine sulphate) is shown for Peaks B, T, M, C and C+ (*n* = 3 ± SD).

The freshwater environmental microbial community was cultured overnight (24 h) to gain insights into potential FOM production by the total microbial community within the laboratory freshwater system. Significant increases in the total fluorescence intensity for all peaks (B, T, C, C+ and M) were seen from the point of inoculation to the 24 h timepoint. The percentage increase in the complete sample fluorescence intensities (without fractionation) during the 24 h incubation for each peak was: Peak B, 1573 ± 54%; Peak T, 1606 ± 90%; Peak M, 4036 ± 1343%; Peak C, 45,315 ± 9095%; Peak C+, 135,707 ± 11,868% (*n* = 3, ±SD). Samples were interrogated to explore the cellular and extracellular nature of the microbially derived FOM. Figure 1 shows the apportionment of the observed fluorescence intensity (_log_QSU) for the fluorescence peaks investigated for all sample fractions, cellular (resuspended and the lysed cell fractions) and extracellular (supernatant), of the microbial community culture.

Peaks B and T were observed in all sample fractions for the microbial community cultures (Figure 1). The Peak B fluorescence intensity in the supernatant increased during the 24 h incubation, but this increase was lower than that detected within the cellular fractions (the resuspended cells and lysed cells). Peak T is also identified in all sample fractions (Figure 1), although the Peak T fluorescence intensity for the supernatant (extracellular) fraction is almost three orders of magnitude lower in comparison to the Peak T intensity for both the resuspended cells fraction and the lysed cells (cellular). Peak C was found in all sample fractions (Figure 1), with the highest Peak C fluorescence intensity observed in the extracellular material (supernatant fraction), in agreement with the monoculture data (presented in Section 3.2). Similarly, the highest intensity of Peak C+ fluorescence is seen within the supernatant sample fractions. Figure 1 also shows that Peak M fluorescence is present in all microbial community culture sample fractions (supernatant, resuspended cells and lysed cells), with the Peak M fluorescence intensities for the supernatant being almost twice those of the intensities detected from the resuspended cells’ and the lysed cells’ sample fractions.

### 3.2. Environmental Bacterial Isolate Monocultures

**Figure 2:**
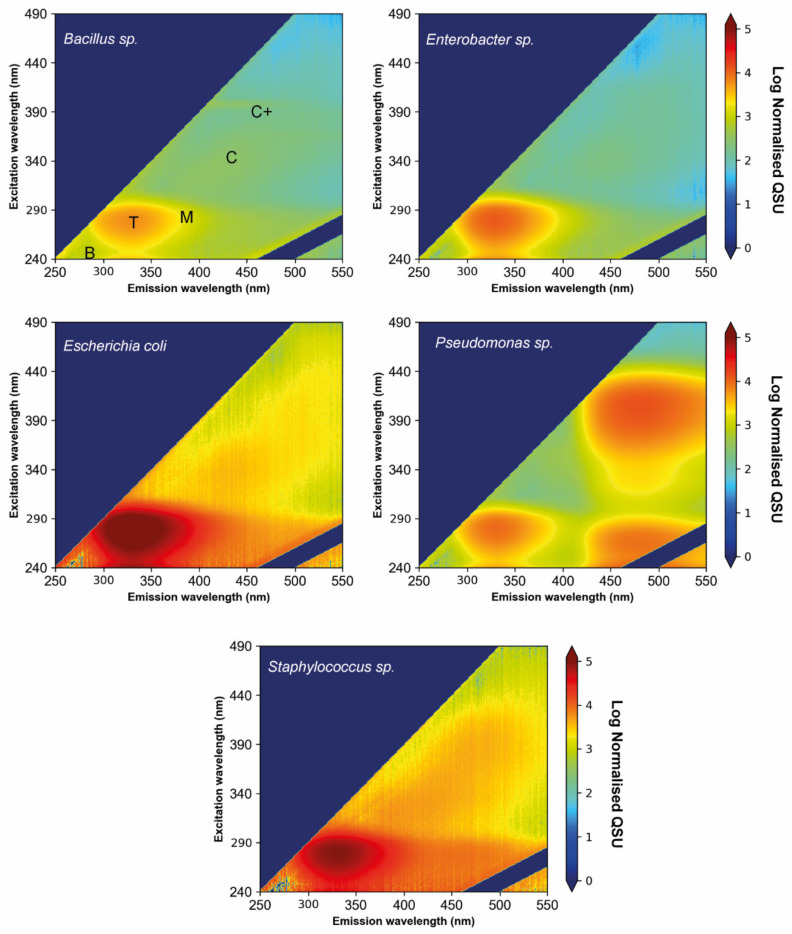
Excitation emission matrices of environmental bacterial isolate monocultures after being grown for 24 h at 30 °C, and prior to fractionation. Fluorescence intensity data reported in ‘Normalised QSU’, whereby cell density normalised QSU is expressed as fluorescence intensity per 10^10^ cfu mL^−1^; data have then been converted to log numbers. All identified fluorescence peaks are labelled and described in Table 1.

The freshwater environmental bacterial isolates were individually cultured overnight (24 h) to obtain a detailed understanding of the potential for different environmental bacterial isolates to contribute to FOM production within environmental freshwater systems. The excitation emission matrices for the complete sample (prior to fractionation) of each of the five bacterial isolates are shown in Figure 2; fluorescence intensity data (QSU) are normalised and corrected for enumeration (detailed in Section 2.5.1). The EEMs for the sample fractions can be found in the Supplementary Materials (Figures S1–S3).

The presence of Peak B within the different investigated monocultures varied considerably. Peak B fluorescence was associated with cellular material within all five environmental bacterial isolates. Peak B fluorescence observed in the complete sample decreased from initial inoculation to 24 h for the *Bacillus* sp., *E. coli* and *Staphylococcus* sp. monocultures (−40.94%, −28.03% and −24.23%, respectively). In contrast, Peak B fluorescence increased for the *Enterobacter* sp. and *Pseudomonas* sp. monocultures (+78.95% and +44.29%, respectively). The largest increase in fluorescence intensities was seen at wavelengths >300 nm, indicating that the observed increases in Peak B for *Enterobacter* sp. and *Pseudomonas* sp. are associated with the presence of high Peak T fluorescence intensities.

**Figure 3:**
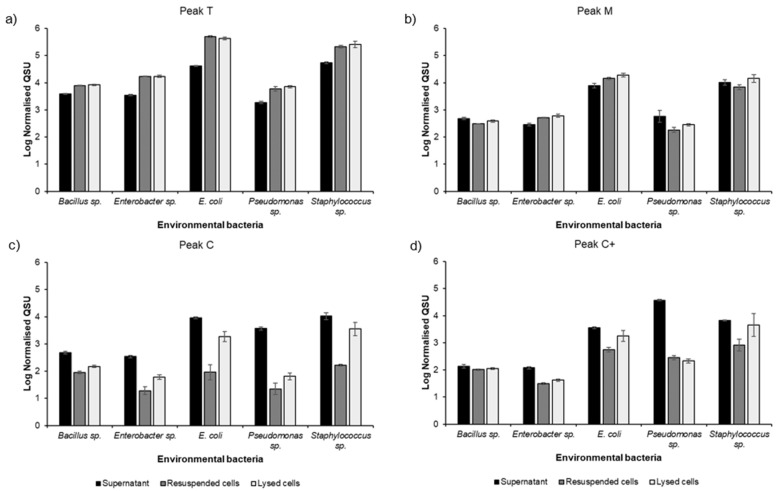
Apportionment of observed fluorescent organic matter peaks T, C, C+ and M in sample fractions of five environmental bacterial isolates, cultured for 24 h at 30 °C. Fluorescence intensities are reported in Log Normalised QSU, whereby cell density normalised QSU is expressed as fluorescence intensity per 10^10^ cfu mL^−1^. (**a**) Peak T, λex/λem 280/330–360 nm; (**b**) Peak M, λex/λem 300 /380–420 nm; (**c**) Peak C, λex/λem 350/420–460 nm; (**d**) Peak C+, λex/λem 400/440–480 nm (*n* = 3 ± SD).

Figure 3 shows the fluorescence intensities of the ubiquitous fluorescence peaks T, C, C+ and M for all five bacterial isolate monocultures. Table 2 shows the total fluorescence associated with the bacterial isolates as the sum of the fluorescence intensities (normalised to cell densities) of peaks T, C, C+ and M, termed the “total relative fluorescence quantum yield”. This table also details the contribution of extracellular (supernatant sample fraction) and cellular (resuspended/lysed cells sample fractions) fluorescence, to the total observed fluorescence for each fluorescent peak (T, C, C+ and M) for each bacterial isolate. This illustrates that *E. coli* and *Staphylococcus* sp. cells exhibit higher total relative fluorescence than *Pseudomonas* sp., *Enterobacter* sp. and *Bacillus* sp.

Peak T fluorescence is seen at high intensities in all sample fractions for all bacterial isolates analysed (Figure 3a). Peak T accounts for >87% of the total fluorescence intensity for *E. coli*, *Staphylococcus* sp., *Enterobacter* sp. and *Bacillus* sp. (Table 3), whereas Peak T contributes <20% to the total fluorescence intensity for *Pseudomonas* sp. due to the high intensity of Peak C+. Peak T is observed as predominantly cellular (resuspended and lysed cells), accounting for between 68 and 92% of Peak T fluorescence intensity for the five bacterial isolates investigated (Table 3). A two-way ANOVA demonstrated that there is a significant difference (*p* < 0.0001) between the Peak T intensity when analysing all fractions (supernatant, resuspended cells and lysed cells) for all five overnight monocultures. Further analysis was undertaken by classifying the resuspended and lysed cell sample fractions as cellular, and the supernatant as extracellular material. Based on this classification, a paired-samples t-test was undertaken to explore the difference between cellular and extracellular FOM for each isolated species. This revealed a significant difference (*p* < 0.05) between cellular (resuspended and lysed cells) and extracellular (supernatant) Peak T across all species (Table 3).

Peak M, shown in Figure 3b, is ubiquitous across all isolated bacterial species investigated and is present in all sample fractions extracted and analysed, albeit at lower fluorescence intensities than Peak T. Peak M contributes between 2 and 6% of the total fluorescence intensities (Table 3) for the isolates studied. The two-way ANOVA confirmed significant differences (*p* < 0.0001) in the Peak M intensity, with a paired t-test revealing significant differences (*p* < 0.05) between extracellular (supernatant) and cellular (resuspended and lysed cells) Peak M for the *Enterobacter* sp. and *E. coli* isolates only.

Peak C fluorescence is pre-dominantly observed within the supernatant sample fraction across all five bacterial isolates cultured here (Figure 3c), with extracellular Peak C contributing to the majority (80–99%) of the Peak C fluorescence (Table 3), in agreement with the environmental microbial community data (see Figure 1). However, the relative contribution of Peak C to the total fluorescence varies between 2 and 7% across the five isolates (Table 3). A two-way ANOVA identified a significant difference (*p* < 0.001) between the Peak C intensity when analysing all fractions (supernatant, resuspended cells and lysed cells) for all five overnight monocultures. A paired-samples t-test revealed a significant difference (*p* < 0.05) between cellular (resuspended and lysed cells) and extracellular (supernatant) Peak C for *Bacillus* sp., *Enterobacter* sp., *E. coli* and *Pseudomonas sp*. Due to variability in the data, no significant difference (*p* > 0.05) between extracellular and cellular Peak C was identified for *Staphylococcus* sp.; Peak C contributes <3.5% of the relative fluorescence for this bacterial isolate (Table 3). The coefficient of variance for the extracellular and cellular Peak C is 31.74% and 59.02%, respectively.

Similarly, Peak C+ exhibits variation, as highlighted Figure 3d and Table 3. The intensity of Peak C+ for *Pseudomonas* sp. contributes 74% of the total fluorescence intensities in which the vast majority of the observed fluorescence is attributed to extracellular material (>99%). The other species also exhibit Peak C+ fluorescence in both cellular and extracellular material albeit significantly lower, <3%, in terms of its overall contribution to the total fluorescence intensity (Table 3). A two-way ANOVA demonstrated a significant difference (*p* < 0.0001) between the Peak C+ intensity for all fractions (supernatant, resuspended cells and lysed cells) from all five bacterial isolate monocultures. A paired t-test revealed a significant difference (*p* < 0.01) between extracellular (supernatant) and cellular (resuspended and lysed cells) Peak C+ for *Enterobacter* sp., *E. coli* and *Pseudomonas sp*. No significant difference (*p* > 0.05) between extracellular and cellular Peak C+ was identified for *Bacillus* sp. and *Staphylococcus* sp., which only contributes <3% of the relative fluorescence for these bacterial isolates (Table 3). For the *Staphylococcus* sp., the lack of a significant difference between extracellular and cellular Peak C+ is likely to be caused by variation within the data, with the coefficient of variance for the cellular Peak C+ being 81.97%.

## 4. Discussion

### 4.1. FOM Production Potential of a Freshwater Environmental Microbial Community

A complex microbial community, cultured from an environmental freshwater source, was subject to overnight culturing and sample fractionation. The laboratory model data presented demonstrate that the freshwater microbial community studied produced a range of FOM in a freshwater laboratory model system. The microbial community data show the in-situ production of FOM and that the origin of this fluorescing material is both within the cellular structure (cellular) and also exported from cells (extracellular).

Peaks B and T have been frequently characterised within a range of aquatic environments [6,38,57,58] and are associated with microbially produced protein-like material [4,8,11,32,59]. These peaks were observed in all sample fractions associated with the freshwater-derived microbial community cultures, shown in Figure 1. Upon detailed exploration of the environmental microbial community sample fractions, Peak B FOM intensity is present at an order of magnitude greater in the resuspended and lysed cells, suggesting that the majority of the Peak B at hour 24 is present as cellular material (either structural or cellular). The presence, and intensity, of Peak B within the laboratory model system studied here is unexpected since it is associated with labile material [8,11,32]. This is, however, in agreement with observations from recent studies of environmental freshwater systems [6,12,60]. High levels of Peak B production in the microbial community culture, not reflected in the monoculture samples, may also be a function of competition and interactions between members of the microbial community. Peak B has long been associated with phytoplankton in marine research [61,62], which could explain the presence of Peak B FOM in the environmental microbial community. To determine the potential sources of Peak B production by freshwater microbial communities, FOM production by other members of the microbial community (e.g., other bacteria, viruses, phytoplankton, fungi or algae) should be explored.

The presence of Peak T is identified in all sample fractions associated with the microbial community cultures (Figure 1). There is little increase in Peak T fluorescence intensities (+2%), over the 24 h time period, within all supernatant samples. As with Peak B, the majority of Peak T fluorescence is observed in the resuspended and lysed cell fractions, indicating the cellular origin of Peak T fluorescence in this laboratory model. This finding challenges recent environmental groundwater research [31], which concludes that Peak T is predominantly associated with extracellular material [8,32,63]. The Peak T data associated with the environmental bacterial isolate monocultures (Table 3) further support this observation.

Peak M is discussed widely in the literature as autochthonous FOM associated with marine biodegraded organic matter [4,13,36,57,64,65]. This FOM has also been observed in nonmarine environments [66], where it has been linked to microbial processing of organic material [8]. Here, Peak M is rapidly produced (within 24 h) by the environmental microbial community within this freshwater study (Figure 1). This demonstrates that Peak M can be both autochthonous, i.e., produced in-situ as shown here, and derived via the degradation of terrestrial humic substances present in the environment [57]. The fluorescence intensity of Peak M is much greater in the supernatant fraction, more than an order of magnitude greater than observed in the resuspended and lysed cell fractions. The prevalence of Peak M in the supernatant for the mixed environmental culture (Figure 1) is in contrast to the observations associated with the monoculture data (Figure 3b), where Peak M is more evenly distributed across extracellular and cellular material (Table 3). A plausible driver is the possible competition for resources or dominance of specific species, owing to the preferential growth conditions used within this model system, which ultimately impacts on the observed FOM composition. It is clear that further work exploring microbial community interactions is required, but this study shows that microbial communities have the potential to impact FOM composition and intensity in freshwater systems and that such FOM characteristics are related to alterations in metabolic activity and subsequent metabolites that are produced.

Peaks C and C+ are associated with high molecular weight, terrestrially derived, allochthonous organic material [15,20,26]. Previous work has shown that microbes can produce Peaks C and C+ in simple matrices [38,39,40]. In this study, for the microbial community cultures, Peak C was found to be predominantly associated with extracellular material. This is in agreement with the data associated with the bacterial isolates (Table 3) and is also in line with recent environmental observations [31]. High Peak C intensities are observed within the microbial community culture samples (Figure 1). This is in contrast to the bacterial isolate data, which show minimal Peak C fluorescence in relation to the overall fluorescence intensities (Table 3). This observation could be influenced by microbial community interactions and the number and composition of microorganism’s present. While further work is needed to better understand the microbial processes that give rise to the production of Peak C, these data demonstrate that a freshwater-derived environmental microbial community can produce and contribute an appreciable amount of autochthonous extracellular Peak C FOM within a laboratory model system. Figure 1 demonstrates that Peak C intensity, unlike the other peaks analysed here, is significantly different (higher) in the lysed cell fraction in comparison to the resuspended cell fraction. This supports the notion that cell lysis is a vector for FOM release into aquatic environments, impacting on the availability of carbon, as recently reported for marine environments [32].

In our system, the majority of Peak C+ FOM produced by the microbial community is extracellular. Previous literature has suggested that Peak C+ fluorescence may be associated with exotoxin production by specific microorganisms [39,67]. We observed that the freshwater microbial community studied here produced this complex Peak C and Peak C+ FOM, demonstrating that autochthonous and allochthonous FOM is part of a dynamic continuum of microbial processing and production within this laboratory freshwater model system.

### 4.2. FOM Production by Environmental Freshwater Bacterial Monocultures

FOM peaks, such as peaks T, C and M, are common across a range of different freshwater systems. To explore the universal presence of this FOM further and to better understand FOM production from a freshwater microbial community, individual environmental bacterial isolates were investigated. The isolated species were cultured from the same freshwater source (sample) as the microbial community and grown, as monocultures, in the same laboratory model and conditions.

The data presented here demonstrate that all of the environmental bacterial isolates studied are capable of producing a range of FOM, in relation to both the location (peak) and intensity. Within this laboratory system, *Staphylococcus* sp. and *E. coli* were shown to produce the most fluorescence per cfu (colony forming unit), see Table 2. Although only five bacterial isolates are investigated here, the relative contributions of the different fluorescence peaks provide a valuable insight into the contributions of individual bacterial species to freshwater FOM (peak composition and fluorescence intensities).

It has been widely postulated that cell lysis and biodegradation are the mechanisms by which higher molecular weight autochthonous material is derived within marine systems [32,37,38,64,68]. The work here suggests that intact and lysed cells exhibit peak C and M fluorescence (see Figure 3) and that variations in observed fluorescence signatures are most likely derived from the metabolic pathways or metabolite production associated with the bacterial species, rather than cell lysis alone [35]. However, the observed fluorescence intensities for both peaks C and M are elevated in the lysed cells, in comparison to the resuspended cells (Figure 3). As discussed previously, this could be related to the disruption of organic material during the physical lysis, demonstrating the ability of lysis to add to the carbon pool [32]. These experiments were undertaken in a closed system, with no interaction between the lysed cells and a viable community. As such, further work is needed to fully explore in-situ interactions between lysed cells and active microbial communities in freshwater environments.

Peak T fluorescence is identified within the FOM produced by all environmental bacterial isolates studied. In all cases, the highest intensities of Peak T were observed in the resuspended and lysed cells (Figure 3a), with >68% of the Peak T being associated with cellular material for all five species (Table 3). This agrees with our previous work using bacterial reference strains, strongly suggesting that its origin and presence is due to microbial structural components and/or cellular constituents. However, this contradicts some recent groundwater research which demonstrated the majority (>90%) of Peak T fluorescence, in a natural groundwater, to be associated with extracellular material [31]. This demonstrates the need for more experimental work regarding impact of residence time on the release of cellular material into the water body, either via metabolic pathways or via cell lysis [69]. The omnipresence of Peak T within this study and its known association with other microorganisms, such as algae [70,71,72], clearly shows that Peak T fluorescence cannot be attributed as an indicator of specific bacterial species, or as a reliable surrogate for bacterial enumeration in complex aquatic microbial communities. This, in itself, explains the observed variations in correlations between Peak T and bacterial enumeration detailed within previous studies in the literature [27,73,74]. Despite this, the data presented within this study, coupled with our previous work [39], highlight the potential application of Peak T fluorescence for monitoring microbial community activity within freshwater systems, and for identifying pollution events whereby nutrients or microbial contamination give rise to increase microbial activity in environmental freshwater systems.

Peak B, similarly to Peak T, is associated with amino acids, specifically tyrosine [8,32,57,75]. This study supports this association due to the cellular nature of Peak B, as well as the classification of Peak B FOM as autochthonous microbially derived proteinaceous material [11,38,59]. The fluorescence intensity of Peak B decreases during the 24 h culture period for the *Bacillus* sp., *E. coli* and *Staphylococcus* sp. monocultures. This could be explained by the highly labile nature of Peak B FOM, meaning it may be rapidly utilised (<24 h) within the closed monoculture laboratory system investigated here. Furthermore, this FOM has been demonstrated to be more vulnerable to quenching effects, such as energy transfer, depolarisation, oxygen quenching and protein conformation [76,77,78,79,80]. Further work to explore specific interactions between environmental freshwater bacteria and Peak B FOM are required to further understand the lability of Peak B and its role in carbon processing throughout the hydrological continuum.

Peak M was ubiquitous within all sample fractions (cellular and extracellular) for all of the environmental bacterial isolates (Figure 3b). Although identified in all sample fractions for all species, the contribution of Peak M is low, <6% of the total fluorescence intensities (Table 3). We present the possibility of freshwater associated bacteria “engineering” Peak M in-situ, i.e., as a structural component of the cell, metabolic by-product and/or functional protein. This develops the current understanding of the origin of Peak M, which has previously been seen as associated with by-products of bio- or photodegraded organic material [36,57,64], or as a humic-like material identified within the marine environment [11,53,81]. The universal nature of Peak M within the bacterial isolate monocultures suggests that this material is potentially produced via metabolic pathways that are common across the five species analysed.

Peak C-related FOM was ubiquitous across all sample fractions for all environmental bacterial isolate monocultures (Figure 3c), although the relative contribution of Peak C fluorescence is low (<7.5%) for all bacterial species studied (Table 3). The majority of Peak C FOM arising from these bacterial species is associated with extracellular material (Table 3), whereby the highest fluorescence intensities were observed within supernatant fractions (Figure 3c), which is also in agreement with recent environmental observations [31]. Peak C is commonly observed in surface freshwaters and is routinely described and defined as being allochthonous in both origin and nature [8]. The data presented here suggest that Peak C-associated FOM can be derived exclusively via microbial processing, albeit in low intensities, and is associated with extracellular material. This demonstrates that freshwater bacteria are capable of producing FOM attributed to Peak C, meaning the FOM can be autochthonous in origin. This compliments other aquatic organic matter research, which has identified the autochthonous production of higher molecular weight metabolites [22,23,37,82,83]. Further work is needed to identify the specific mechanisms responsible for the export of this extracellular Peak C, and the significance of this contribution in real-world freshwater systems are yet to be determined. This discovery also raises the following questions: (1) whether Peak C FOM observed in freshwater systems is entirely allochthonous material in nature, as is currently assumed within the literature; (2) what is the relative microbial contribution of Peak C FOM; and (3) if this impacts the chemical composition and, therefore, the lability of this Peak C-associated material?

High fluorescence intensity of Peak C+ was identified within the supernatant sample fraction for *Pseudomonas* sp. (Figure 3d), with extracellular Peak C+ accounting for over 99% of the observed Peak C+ fluorescence for this bacterial isolate (Table 3). The dominance of Peak C+ for the environmentally isolated *Pseudomonas* sp. suggests that Peak C+ fluorescence is related to specific processes and/or metabolites which have specific biological functions for this bacterial species. For example, previous work has associated the production of Peak C+ fluorescence by *Pseudomonas aeruginosa* (NCIMB 8296) with the siderophore pyoverdine, a high molecular weight (1365 Da) extracellular iron-scavenging metabolite [39,67]. Further work exploring the metabolic mechanisms that are responsible for Peak C+ production may aid in the understanding of what the presence of this FOM can inform us on regarding freshwater processes. For instance, if Peak C+ FOM production is related to exotoxin production due to nutrient deficits, such as iron or phosphate, this could be used to infer ecosystem function and/or chemical water quality.

The FOM data from the bacterial isolates clearly demonstrate that freshwater bacteria can produce a range of FOM, including fluorescent compounds associated with higher molecular weight [20,35,36,84,85], previously thought to be of terrestrial origin as opposed to microbial origin [13,14,57]. This establishes environmental freshwater bacteria as “engineers” of aquatic FOM associated with both low and high molecular weight compounds within the laboratory models used in this study. This raises the question as to the extent to which such microbial processing leads to the freshwater FOM characteristics observed in environmental freshwater systems. It also challenges the binary classification of freshwater FOM as either allochthonous or autochthonous, suggesting that FOM processing and production occurs along a dynamic continuum.

### 4.3. Future Work

The use of a laboratory model presents the opportunity to interrogate culturable environmentally sourced bacterial isolates and communities in controlled conditions. This approach offers insights into FOM production by these microorganisms, albeit with limitations. These limitations include the role and impact of uncultured bacteria on the FOM signature and composition. To further the understanding of microbial–FOM interactions, a more representative laboratory model could be employed with the aim of limiting preferential growth conditions, for example by using a simulated freshwater matrix instead of a growth media. Alongside this, the continued use of different environmentally derived freshwater microorganisms, including the exploration of algae, fungi, viruses and other bacteria, would greatly enhance the current knowledge base surrounding microbial interactions with freshwater FOM and how this impacts global biogeochemical cycling.

In addition to the development of more representative model systems, further understanding of FOM production and transformation could be gained through the use of high-resolution analytical techniques, such as FTICR-MS, Orbitrap-MS or metabolomics, in tandem with fluorescence spectroscopy. The use of such techniques, specifically FTICR-MS, is used for the quantification of FOM components that have been identified and derived from parallel factor (PARAFAC) analysis [4]. However, much of this work has been associated with highly complex environmental samples [4,5,25]. Employing these quantitative techniques within exploratory laboratory model systems could provide valuable insights into the characteristics and composition of FOM and DOM as well as making it possible to ascertain the biological processes that give rise to specific FOM production.

## 5. Conclusions

This work has, at the very least, questioned the binary classification of allochthonous or autochthonous FOM as currently applied to freshwater systems. Environmental freshwater microbes produce a range of FOM in-situ (including assumed complex high molecular weight material), both processing and producing FOM along a dynamic continuum, albeit demonstrated in a laboratory freshwater model system. The microbial production and potential export of metabolic by-products and/or the production of functional proteins are possible reasons for the observed FOM characteristics. Understanding the mechanisms for this will provide knowledge and insight regarding microbial processing and its relevance to carbon cycling on a global scale.

This work explicitly explores the potential contribution of microbial FOM production and the impact of cell lysis on the composition and intensity of freshwater FOM. The lysed cell fractions exhibit similar fluorescence characteristics to intact cells, but do demonstrate elevated fluorescence intensity in the lysed cells fraction for both peaks C and M. This demonstrates that cell lysis can contribute to FOM and the carbon pool, but is unlikely to be the sole source of ‘microbial’ Peak C and Peak M. However, further work regarding the role cell lysis plays in freshwater FOM transformation and carbon cycling in dynamic active environmental microbial communities, and over time, is needed.

This study identifies the production of Peak B by the environmental microbial community, but this is not reflected by the individual bacterial isolates. Interactions within the microbial community, or community members (not isolated here), could be responsible for the Peak B FOM signatures seen. This may also be explained by the use of bacterial monocultures here, something that does not exist in nature, which may have both produced and processed Peak B FOM, thereby rapidly preventing the identification of this labile FOM after the 24 h culturing period. The frequent, but not universal, presence of Peak B in environmental freshwater systems highlights further exploration of this phenomenon as essential for understanding labile FOM interaction and the potential impacts this may have on the global carbon cycle.

This work confirms the microbial origin of Peak T. The presence of Peak T fluorescence is ubiquitous in all sample fractions, being identified at significantly higher fluorescence intensities within cellular sample fractions. Further work is required to understand the mechanisms by which this FOM is produced and exported into freshwater systems. Peak C and M fluorescence are universally produced within all sample fractions, but Peak C is predominantly found in the supernatant sample fraction. This suggests that the majority of Peak C is exported as extracellular material into the surrounding system. These results support the findings of previous work that investigated bacterial reference strains. Importantly, this work demonstrates that environmental freshwater microbes also produce and export a range of FOM. This knowledge could have implications for enhancing our understanding of the role that freshwater systems play in the global cycling of carbon.

## Figures and Tables

**Table 1:** Fluorescence peaks produced and identified from the overnight cultured environmental microbial community and environmental bacterial isolate cultures.

λ_ex_/λ_em_ (nm)	Named Fluorescence Peak	Conventional Descriptions
250/290–320	B	Protein-like material, autochthonous, resembles tyrosine fluorescence, associated with amino acids
280/320–360	T	Protein-like material, autochthonous, resembles tryptophan fluorescence, associated with microbial processes
300/380–420	M	Humic-like material, autochthonous, associated with microbial degradation
345/420–460	C	Humic-like material, allochthonous, terrestrial in origin, resembles humic acids
400/440–490	C+	Humic-like material, allochthonous, terrestrial in origin, resembles humic acids

Nomenclature and association derived from Coble et al. (2014) and Fox et al. (2017).

**Table 2:** Total relative fluorescence quantum yield for five environmental bacterial isolates (isolated from an environmental freshwater sample) after growth for 24 h at 30 °C; total fluorescence quantum yield is determined from the summative fluorescence intensities (normalised to cell densities) for peaks T, C, C+ and M, normalised to the highest intensity. The percentage of the total calculated fluorescence which is attributed to extracellular material (supernatant fraction) and cellular material (resuspended and lysed cells) is also shown.

Bacterial Isolates	Fluorescence Peaks (T, C, C+ & M)		
	Total Relative Fluorescence Quantum Yield (%)	% Extracellular	% Cellular
*E. coli*	**100.00**	11.46	88.54
*Staphylococcus* sp.	**68.95**	30.97	69.03
*Pseudomonas* sp.	**9.25**	85.91	14.09
*Enterobacter* sp.	**4.04**	19.21	80.79
*Bacillus* sp.	**2.50**	36.27	63.73

**Table 3:** Relative contribution (%) of peaks T, C, C+ and M to fluorescence intensities (determined from the summative cell density normalised fluorescence intensities of these peaks) produced by environmental bacterial isolates, cultured for 24 h at 30 °C. The percentage fluorescence intensities for each fluorescence peak attributed to extracellular material (supernatant fraction) and cellular material (both the resuspended and lysed cells) for all isolates is also shown.

		Bacterial Isolates				
Fluorescence Peaks		*E. coli*	*Staphylococcus* sp.	*Pseudomonas* sp.	*Enterobacter* sp.	*Bacillus* sp.
**Peak T**	**Relative contribution %**	**92.71**	**88.12**	**16.77**	**93.66**	**87.69**
	% Extracellular	8.24	26.50	22.28	16.85	32.16
	% Cellular	91.76	73.50	77.72	83.15	67.84
**Peak M**	**Relative contribution %**	**4.52**	**5.69**	**1.71**	**3.86**	**6.14**
	% Extracellular	31.83	49.04	70.41	33.53	58.26
	% Cellular	68.17	50.96	29.59	66.47	41.74
**Peak C**	**Relative contribution %**	**1.88**	**3.45**	**7.40**	**1.76**	**4.36**
	% Extracellular	89.94	83.35	98.76	89.66	79.51
	% Cellular	10.06	16.65	1.24	10.34	20.49
**Peak C+**	**Relative contribution %**	**0.89**	**2.74**	**74.12**	**0.72**	**1.81**
	% Extracellular	74.78	68.23	99.32	76.47	55.53
	% Cellular	25.22	31.77	0.68	23.53	44.47

## Data Availability

Publicly available datasets were analysed in this study. These data can be found here: [http://researchdata.uwe.ac.uk/629].

## Supplementary Materials

The following are available online at https://www.mdpi.com/article/10.3390/microorganisms9081623/s1, Table S1: Physicochemical parameters for the environmental freshwater sample used to obtain both the environmental community and bacterial isolates, Figure S1: Enumeration normalised excitation emission matrices of the supernatant sample fraction of the 24-h environmentally sourced monoculture samples, Figure S2: Enumeration normalised excitation emission matrices of the resuspended cells sample fraction of the 24-h environmentally sourced monoculture samples, Figure S3: Enumeration normalised excitation emission matrices of the lysed cells sample fraction of the 24-h environmentally sourced monoculture samples.
