# Supplementary material for: Laboratory In-Situ Production of Autochthonous and Allochthonous Fluorescent Organic Matter by Freshwater Bacteria
Source: Microorganisms. 2021 Jul 29;9(8):1623. doi: 10.3390/microorganisms9081623 (PMC8400322; doi:10.3390/microorganisms9081623)

Supplementary Material

Table S1. Table of data for the physicochemical parameters for the envirnmental freshwater sample used to obtain both the environmental community and bacterial isolates. The freshwater sample was collected on 01/06/2017 at 13:55 BST. The table shows data collected via an *in situ* water quality sensor (Eureka Manta 2, RS Hydro, UK) and data from an accredited off-site laboratory (UK accredited laboratory, ISO 17025 (2005)).

	Parameter	Unit	Value
***In situ* sensor data**	Temperature	(°C)	18.3
	pH		7.89
	Dissolved Oxygen	(mg/L)	6.61
	Conductivity	(µS/cm)	595.9
**UK accredited laboratory data**	pH		7.9
	Conductivity	(µS/cm @ 20°C)	537
	Turbidity	NTU	44
	Colour (spectrophotometer)		7
	Nitrate (calculated)	mg/L NO3	4.7
	Nitrite	mg/L NO2	0.149
	Orthophosphate	mg/L P	0.63
	Sulphate	mg/L	96
	Chloride	mg/L	43
	2 day plate count @ 37	cfu/mL	6700
	3 day plate count @ 22	cfu/mL	13400
	Total coliforms	cfu/100 mL	860
	E. coli	cfu/100 mL	270
	Clostridium perfringens	cfu/100 mL	340
	Enterococci	cfu/100 mL	120
	P. aeruginosa	cfu/100 mL	1000

Figure S1. Enumeration normalised excitation-emission matrices of the supernatant sample fraction of the 24-hour environmentally-sourced monoculture samples. Samples were incubated at 30°C. Colour bars are quinine sulphate units, $_{log}$QSU per cfu mL^{-1}, where 1 QSU = 1 µg L^{-1} quinine sulphate. Fluorescence peaks identified from the environmental isolate bacterial cultures are labelled and described in Table 1.

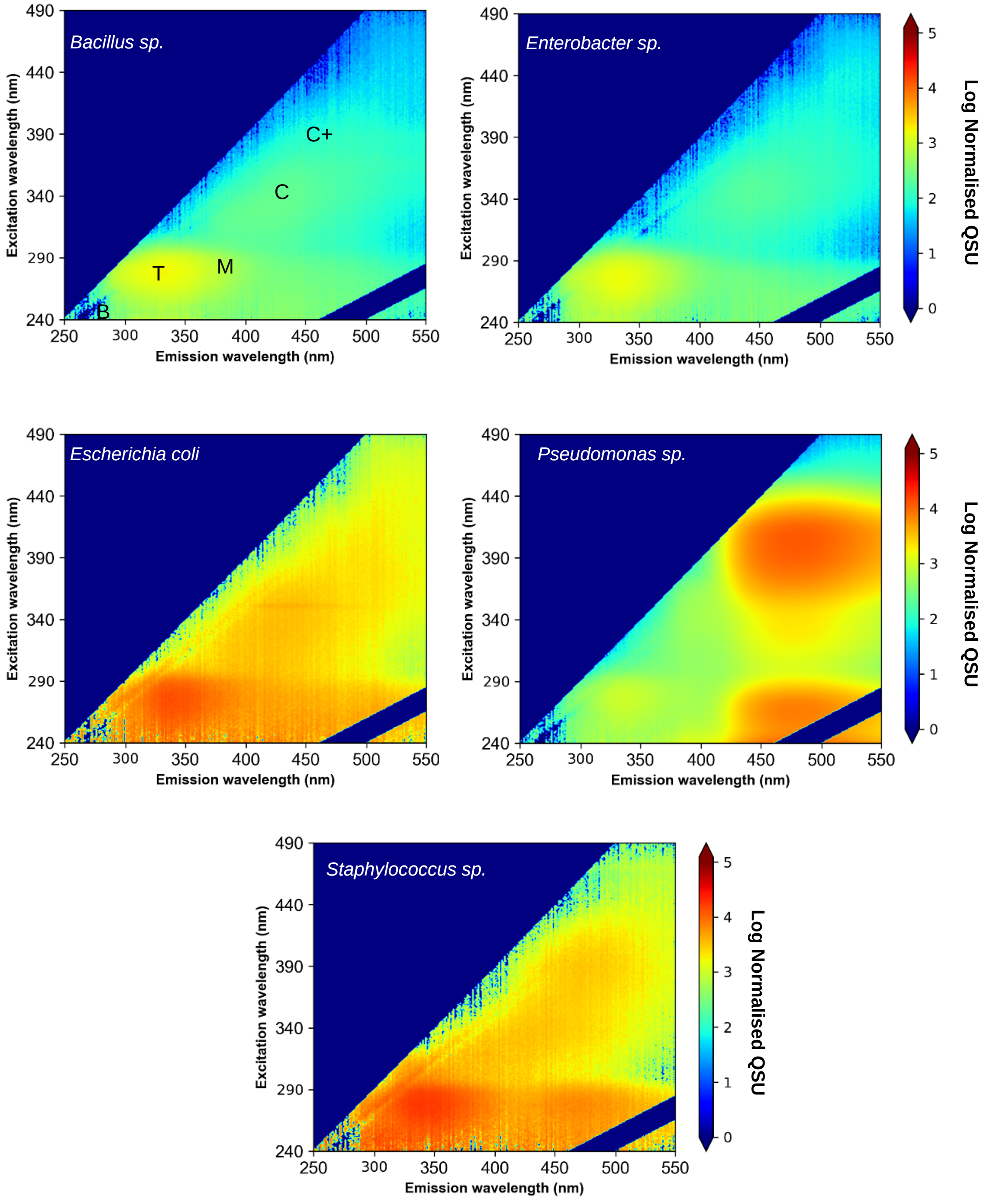

Figure S2. Enumeration normalised excitation-emission matrices of the resuspended cells sample fraction of the 24-hour environmentally-sourced monoculture samples. Samples were incubated at 30°C. Colour bars are quinine sulphate units, $_{log}$QSU per cfu mL^{-1}, where 1 QSU = 1 µg L^{-1} quinine sulphate. Fluorescence peaks identified from the environmental isolate bacterial cultures are labelled and described in Table 1.

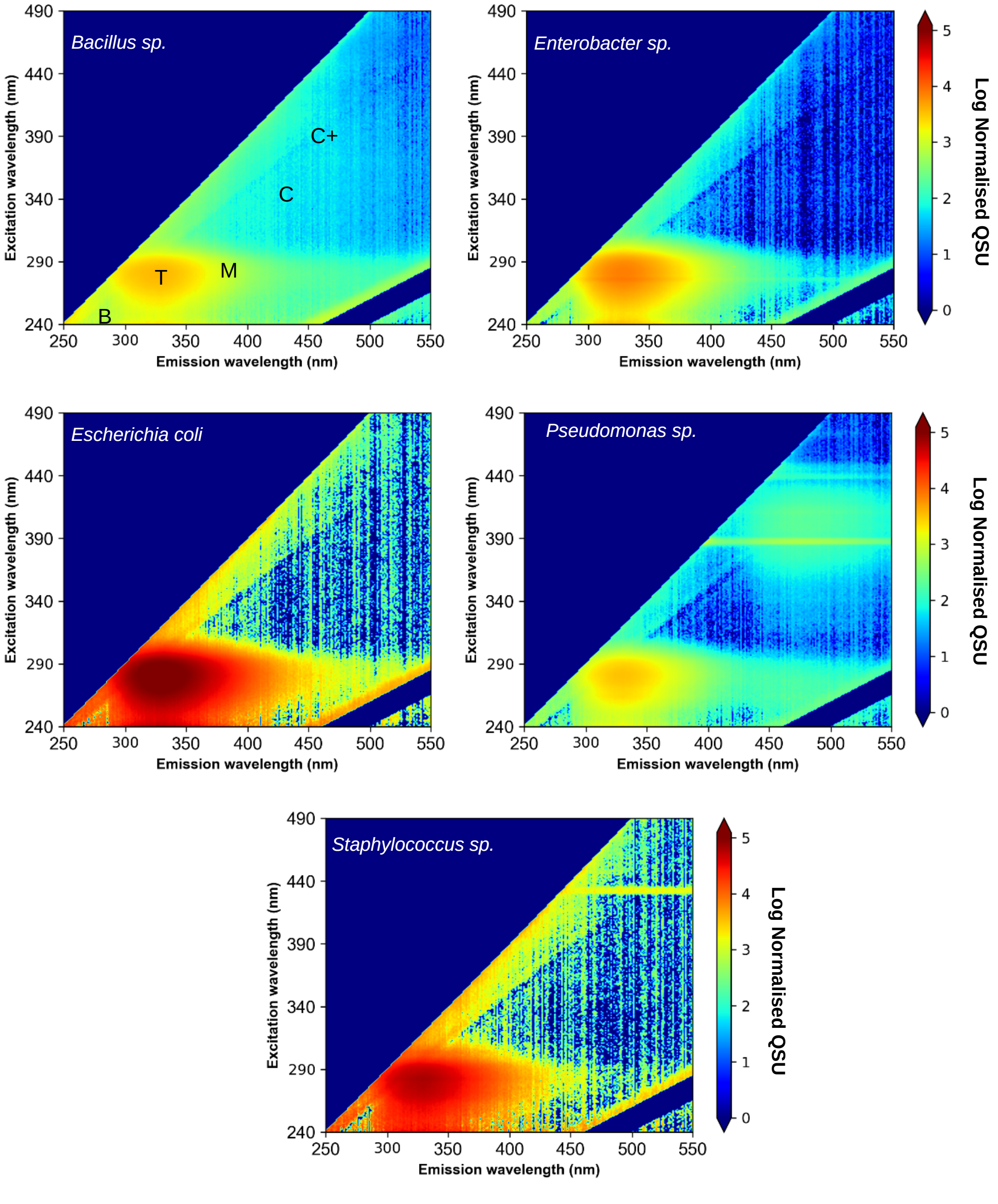

Figure S3. Enumeration normalised excitation-emission matrices of the lysed cells sample fraction of the 24-hour environmentally-sourced monoculture samples. Samples were incubated at 30°C. Colour bars are quinine sulphate units, $_{log}$QSU per cfu mL^{-1}, where 1 QSU = 1 μg L^{-1} quinine sulphate. Fluorescence peaks identified from the environmental isolate bacterial cultures are labelled and described in Table 1.

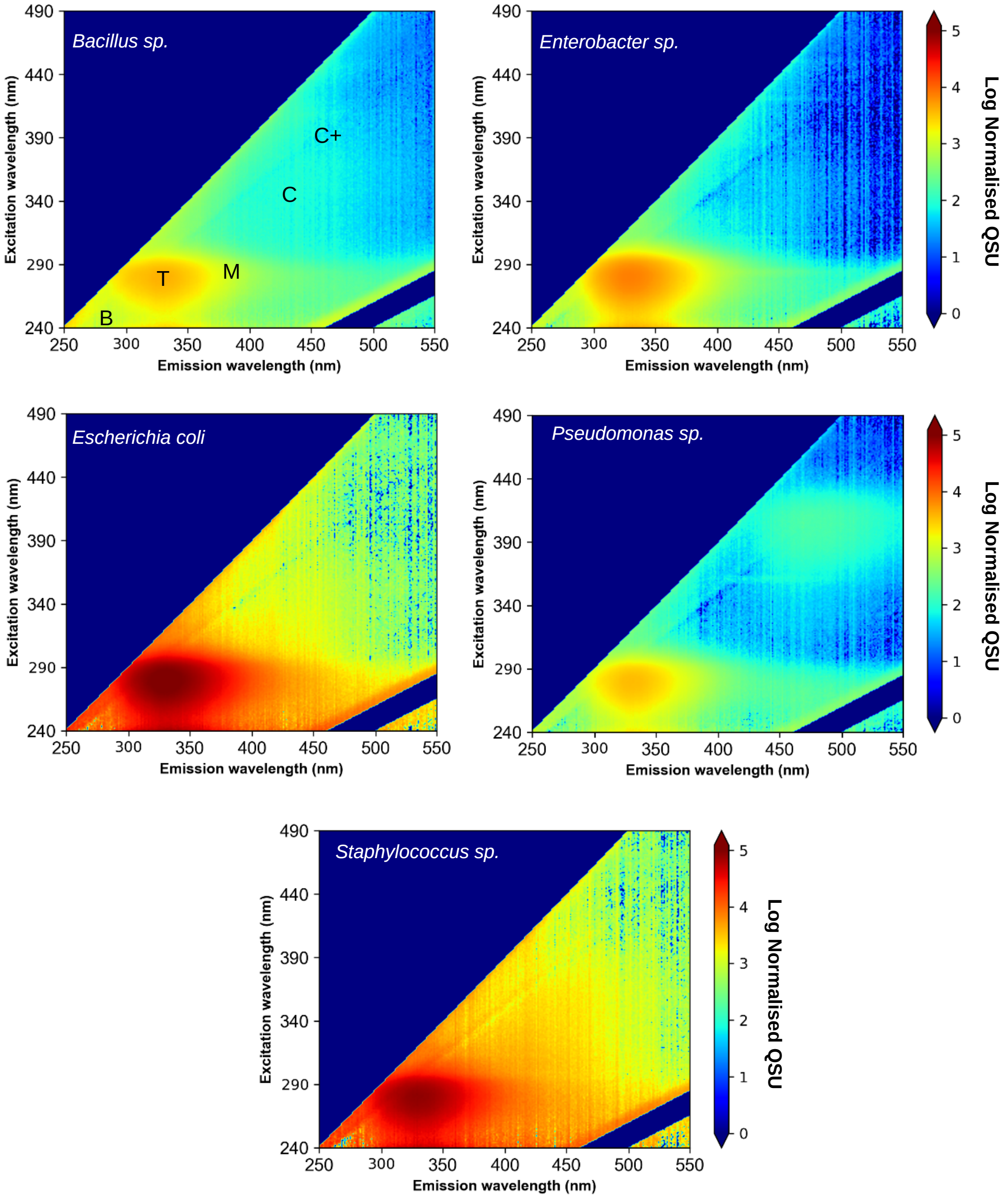

Supplement: Supplementary file 1 [file microorganisms-09-01623-s001.zip › microorganisms-1279960-supplementary.pdf]
